# Clinical and radiological outcomes of using locked intramedullary nails in the treatment of severe frontal plane lower limb deformity in adolescents with hypophosphatemic rickets (mid-term results)

**DOI:** 10.1007/s00402-026-06313-4

**Published:** 2026-05-04

**Authors:** Sherif Galal, Al-Munqith Al-Abri, Ghassan Al-Habsi, Mohammed Al Ghammari, Ahmed Elissawi, Wessam Gamal Abou Senna, Amr Said Arafa

**Affiliations:** 1 Khoula Hospital, Muscat, Oman; 2 Cairo University, Giza, Egypt; 3 Sultan Qaboos University, Muscat, Oman

**Keywords:** Deformity Correction, X-Linked Hypophosphatmeis, Rickets, Intrameduallry Nailingm XLHPR

## Abstract

**Introduction:**

Lower limb deformities in patients with hypophosphatemic rickets are multi-apical and require multiple osteotomies for correction. Intramedullary nails (IMNs) are used to fix multiple osteotomies. Authors of this studyaimed to assess the clinical and radiographic outcomes of using IMNs for correcting lower limb deformity in adolescents with hypophosphatemic rickets.

**Methods:**

This prospective study included patients with hypophosphatemic rickets who underwent deformity correction between November 2020 and November 2023 using IMNs, with a minimum follow-up of 2 years. Clinical outcomes were assessed using the Lower Limb Deformity-Scoliosis Research Society (LD-SRS) score. Radiographic outcomes measured included the mechanical tibiofemoral angle (*mTFA*), mechanical axis deviation (MAD), mechanical lateral distal femoral angle (*mLDFA*), mechanical medial proximal tibial angle (*mMPTA*), and Stevens’ knee joint zoning.

**Results:**

Twenty patients (25 limbs and 35 bones) were included (mean age: 16 years; range: 13–22 years). The LD-SRS score improved from 3.2 ± 0.4 preoperatively to 4.3 ± 0.7 postoperatively. Preoperative mechanical tibiofemoral angle was 27.8° ± 12.1° and 17.33° ± 7.9° in the varus and valgus groups, respectively, improving to 3.4° ± 6.4° and 2° ± 5.2°, respectively. Preoperative mechanical axis deviation was 82.2 ± 35.6 mm and 41.8 ± 22.2 mm in the varus and valgus groups, respectively, improving to 9.2 ± 20.1 mm and 4.6 ± 12.6 mm, respectively. Preoperative mechanical lateral distal femoral angle was 102.4° ± 9.7° and 77.9° ± 9.8° in the varus and valgus groups, respectively, improving to 89.9° ± 3.1° and 88.1° ± 3.1°, respectively. Preoperative mechanical medial proximal tibial angle was 78.7° ± 8° and 89.7° ± 3.9° in the varus and valgus groups, respectively, improving to 88.6° ± 3.4° and 88.2° ± 2.9°, respectively. Preoperative Stevens’ knee joint zoning was Zone 3 in all patients, improving to Zone 1 in 16 limbs, Zone 2 in eight limbs, and Zone 3 in one limb.

**Conclusion:**

Correction of severe frontal plane lower limb deformities in adolescents with hypophosphatemic rickets using IMNs yields good clinical and radiographic outcomes at 2-year follow-up.

**Supplementary Information:**

The online version contains supplementary material available at 10.1007/s00402-026-06313-4.

## Introduction

### Background

X-linked hypophosphatemic rickets (X-LHPR) is a rare metabolic disorder associated with progressive rickets and profound limb deformities, first described by Albright et al. [1] in 1937. Gene mutations leading to the dysregulation of fibroblast growth factor 23 result in chronic renal phosphate wasting and impaired activation of 1,25-dihydroxyvitamin D [2, 3].

Early radiological findings, typically noticeable after 1 to 2 years of age, include widened and irregular physes, cupped and flared metaphyses, and generalized osteopenia. Patients usually present with delayed linear growth and lower limb deformities, most commonly around the knees. Generally, deformities are bilateral and manifest as genu varum, genu valgum, or windswept deformity [4–7].

The conventional treatment for X-LHPR involves pharmacologic supplementation with phosphate and vitamin D analogs to reverse or prevent further deformities [5, 8]. Burosumab, an anti-fibroblast growth factor 23 antibody, has been approved for treating this disease and is expected to improve bone deformities [9–11].

Surgical treatment is indicated for patients with severe or progressive deformities that persist despite optimal pharmacologic management [5]. Several surgical methods, including guided growth and corrective osteotomy with external or internal fixation, can effectively restore neutral lower limb alignment [12]. Guided growth has gained popularity, as it allows for deformity correction before substantial diaphyseal abnormalities develop. However, this approach corrects only frontal plane deformities without addressing the torsional component and rebound deformity after plate removal has been reported [13]. Patients with limited remaining growth potential, typically near puberty, often develop multi-apical complex deformities requiring multiple osteotomies for correction. Fixation is particularly challenging in soft bone. Intramedullary nails (IMNs) are advantageous in such cases because they provide stable fixation across multiple osteotomy sites and span the entire bone length, making them ideal for structurally weak bone.

### Objective

The aim of this study was to assess the clinical and radiographic outcomes of using IMNs for correcting lower limb deformity in adolescents with hypophosphatemic rickets, with the hypothesis that this approach yields good clinical and radiological outcomes.

## Methods

### Study design

A single-center prospective cohort study was conducted between November 2020 and November 2023.

### Setting

After obtaining approval from the research ethics committee, this study was conducted at a public health service Level 1 referral center.

### Participants

The study included all patients with a confirmed diagnosis of hypophosphatemic rickets (made by the endocrine department) who had single or multilevel lower limb frontal plane deformity requiring correction. Exclusion criteria were non-documented diagnosis of hypophosphatemic rickets, closed physes (or less than 2 years of growth remaining), less than 1-year of regular oral medication for hypophosphatemic rickets, preoperative blood phosphorus value of less than 2.5 mg/dL. Preoperative evaluation included a clinical examination, anteroposterior and lateral views of both the femora and tibias and standing full-length radiographs of both lower limbs. Radiographs were also obtained at the final follow-up visit. If a rotational deformity was clinically detected, a computed tomography (CT) rotational profile was also obtained.

A preoperative deformity analysis was performed according to the center of rotation of angulation (CORA) method described by Paley et al. [14]. Using anatomical axis planning, the apex of the deformity was identified by drawing the anatomical axis line representing the proximal and distal bone segments. If these two axes were found to intersect outside the bone or away from the level of the obvious deformity, this indicated the presence of a second deformity apex (CORA), and a third axis line was drawn to represent the anatomical (mid-diaphyseal) axis of the middle segment. The intersections of this third line with the proximal and distal segment lines represented separate apices (two CORAs). Based on the osteotomy guidelines described by Paley et al. [14], if the osteotomy was performed at the level of the apex, only angulation was needed; however, if the osteotomy was performed away from the apex, mandatory translation was required to accurately correct the deformity. The goal of deformity correction was to achieve neutral limb alignment with no mechanical axis deviation (MAD); that is, ensuring that the limb mechanical axis line passed through the center of the knee after correction. It was noted that patients were of short stature, requiring surgeons to be prepared with shorter nail lengths, which may not always be available in standard trauma nail sets. Moreover, their canal was not wide and would accommodate nails with a diameter of 9–10 mm only.

### Intervention and surgical technique

The entire procedure was performed using a fixator-assisted nailing (FAN) technique [15]. Osteotomy around the knee was required in most cases since most deformities had an apex around the knee.

**Fig. 1:**
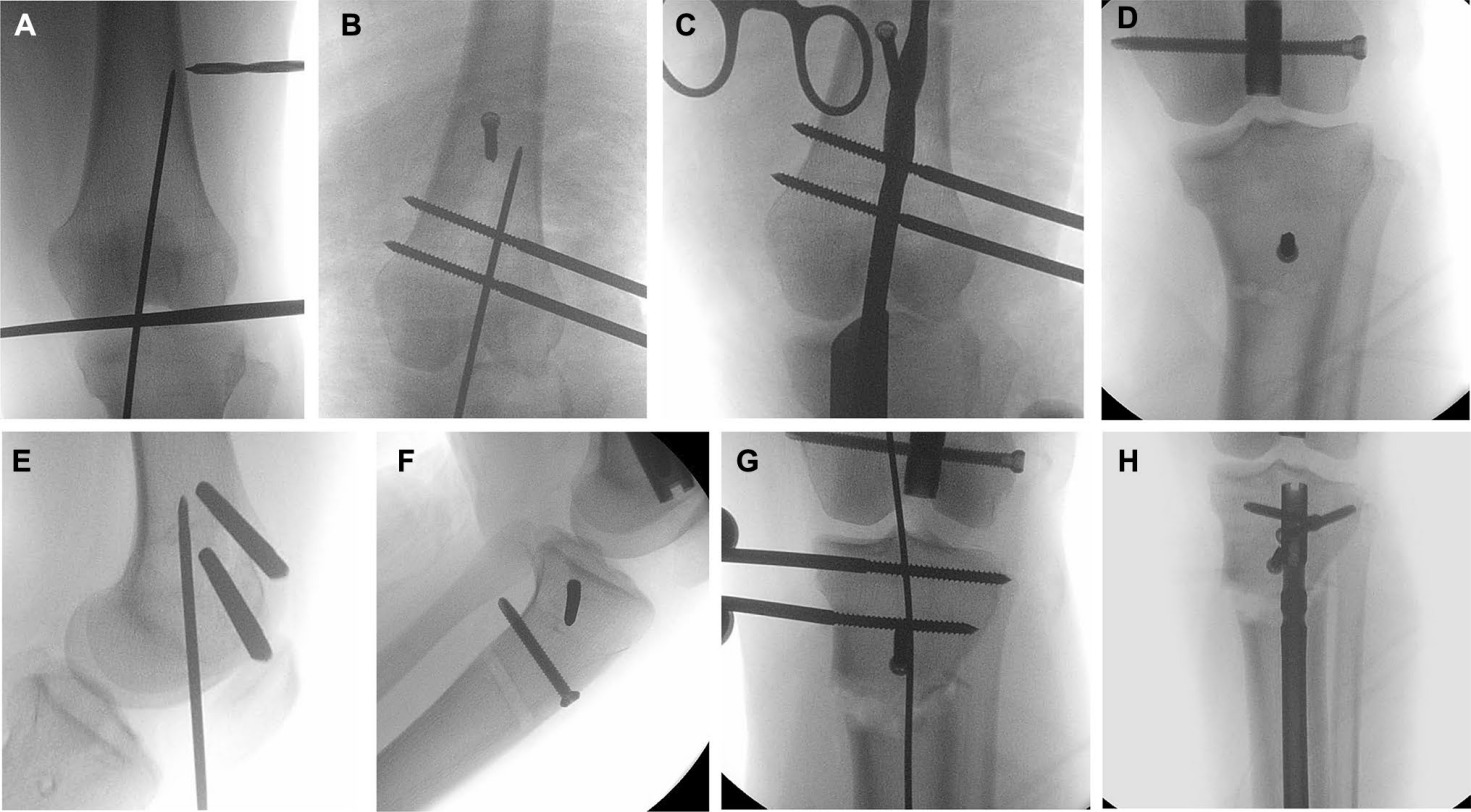
Surgical technique; **A**: determining osteotomy level, **B**: starting guide wire along the nail trajectory (anatomical axis) in metaphyseal segment with 2 Schanz pins perpendicular to the nail trajectory, **C**: starting reamer to establish nail path in metaphyseal segment directed between the blocking screw and the cortex; **D**: blocking screw in metaphyseal segment to direct the nail passage and limit nail play in this wider segment. **E**: Schanz pins are anterior to the starting wire and the nail path in the metaphyseal segment in the femur. **F**: Schanz pins are posterior to the starting wire and the nail path in the metaphyseal segment in the tibia. **G**: Schanz pins are used to joystick the metaphyseal segment and hold any necessary translating till the regular guide wire is passed down the medullary canal. **H**: The nail is passed and locked, and the fixator is removed

During preoperative planning, the surgeon identified where the anatomical axis (nail path) in the metaphyseal segment intersected the cortex toward the diaphysis and measured the distance from this point to a recognizable radiographic landmark, such as the knee joint line (Fig. 1a). This allowed the surgeon to easily replicate this line intraoperatively by inserting a guide pin (Fig. 1b) from the knee’s entry point, directing it to the identified point on the cortex. Canal venting was performed using multiple drill holes at the planned osteotomy site before reaming. A starting reamer was passed along the guide pin in the metaphyseal fragment to create the nail path, but it stopped short of the planned osteotomy site (Fig. 1c) [16], thus creating a nail path that matched the anatomical axis of the metaphyseal segment. With the starting reamer still inside, blocking screws (Fig. 1d) [17] were inserted to prevent nail play.

Successive reaming of this segment was performed until the planned canal size was achieved. Two Schanz pins were inserted perpendicular to the guide pin (nail path) in the frontal plane (Fig. 1b) but positioned anterior to the nail tract in the femur (Fig. 1e) and posterior to the nail tract in the tibia (Fig. 1f), ensuring clearance from the path of the reamers and nail. Another Schanz pin was inserted proximally, above the nail (in the femur), or distally, below the nail (in the tibia), perpendicular to the shaft and anatomical axis of the diaphyseal segment in the frontal plane.

When placing the pins, care was taken to ensure they were inserted orthogonally to the corresponding segment in the axial plane. This was achieved by passing the pins located near the knee parallel to the ground while the knee was in neutral rotation (patella-forward position), the pins near the hip while the hip was in neutral rotation (mid-rotation arc), and those near the ankle while the ankle was in neutral rotation. Thus, when the pins were brought parallel to each other and connected to the fixator after osteotomy, both frontal and rotational deformities were corrected.

It is also worth noting that femoral deformities are usually multiapical in the frontal plane, with greater sagittal femoral bowing, which is corrected with nail passage. The authors of this study also recommend correcting any rotational deformity after nail passage and before locking the nail, as correcting the rotation with the nail inside prevents any undesired translation at the osteotomy sites that may hinder nail passage.

Percutaneous osteotomy (using a small osteotome) was performed at the planned site. If translation at the osteotomy site was needed, it was performed using the proximal and distal Schanz pins as joysticks to manipulate bone fragments (Fig. 1g) or with the help of a small osteotome pushed across the osteotomy site. The fixator was locked, and limb alignment was checked using an alignment rod. A blunt-tipped intramedullary guidewire for the nail reamers was then inserted along the pre-prepared canal of the metaphyseal fragment and further along the medullary canal (Fig. 1g). Reaming continued until the planned canal diameter was achieved. An IMN was then inserted (Fig. 1h). The final alignment was checked again; more blocking screws were added if needed to ensure that no loss of position occurred when the temporary fixator was removed. In the leg (during tibial correction), prophylactic fasciotomy of the lateral compartment (when performing fibular osteotomy) and subcutaneous fasciotomy of the anterior compartment [18] were performed to avoid compartment syndrome, which may occur with acute deformity correction.

During surgery, maintaining the patient warm by administering intravenous warm isotonic sodium chloride solution helped preserve blood flow to the operated limb and counter any vascular spasms or stretches that may result from acute deformity correction.

### Postoperatively

Patients were permitted to bear weight once they were comfortable to (governed by their pain level following surgery) and were observed after 2 weeks for a wound check and then monthly with radiographs (anteroposterior and lateral views of the femur and/or tibia) to assess union (defined as the presence of three continuous cortices). Full-length radiographs of both lower limbs were obtained at the final follow-up. All patients were followed up for a minimum of 2 years.

### Outcomes and sample size

The 2-year postoperative Lower Limb Deformity-Scoliosis Research Society (LD-SRS) score [19] was used as the primary outcome measure and was obtained before surgery and at the final follow-up visit. Secondary outcome measures included radiographic parameters, such as the knee joint zoning method described by Stevens [20], the mechanical tibiofemoral angle (mTFA) , mechanical axis deviation (MAD), mechanical medial proximal tibial angle (mMPTA), mechanical lateral distal femoral angle (mLDFA), knee range of motion and complications.

All radiographic measurements were performed by a limb reconstruction and complex deformity fellowship-trained orthopedic surgeon.

The final correction was evaluated using the knee joint zoning method. If the mechanical alignment of the lower limb passed through the knee joint in Zone 1, the surgical outcome was considered excellent; if it passed through Zone 2, it was considered good; and if it passed through Zone 3, it was considered poor.

The LD-SRS score assesses 30 questions covering five domains: satisfaction with treatment, pain, function, mental health, and self-image. Each answer is scored from one (worst) to five (best), and a final score from one (worst) to five (best) is calculated by dividing the sum of all answers by the number of answered questions. A minimum clinically important difference of 10% (0.5 points) was set, as smaller differences cannot be considered clinically significant. In our sample size analysis, the standard deviation of the LD-SRS score (0.6 points) was used based on previous reports [19]. With a significance level of 5% (two-sided confidence interval of 95%) and 90% power, the sample size was calculated to include at least 30 procedures.

### Statistical analysis

Data were coded and entered using the Statistical Package for the Social Sciences version 28 (IBM Corp., Armonk, NY, USA). Data were summarized using the mean, standard deviation, median, minimum, and maximum for quantitative data, and frequency (count) and relative frequency (percentage) for categorical data. Comparisons between pre- and postoperative data were performed using a paired t-test for normally distributed quantitative variables and non-parametric Wilcoxon signed-rank test for non-normally distributed quantitative variables Statistical significance was set at *p* < 0.05 [21].

### Ethics and funding

This study was approved by the institutional review board. Informed consent was obtained from each patient before inclusion in the study. The authors declare no conflicts of interest. No benefits in any form were received or will be received from any commercial party directly or indirectly related to the subject of this study. This study did not receive any funding.

## Results

Twenty patients (25 limbs, 35 procedures) were enrolled to accommodate any loss during the follow-up. The patients’ age was 14.80 ± 2.19 years (median 16, range 13–22 years); 10 patients were women (50%), and 10 patients were men (50%). The right lower limb was involved in 15 patients (60%), and the left lower limb in 10 patients (40%) (Table 1). Twenty patients underwent 35 procedures, including femoral osteotomy alone in 10 limbs (40%), tibial osteotomy alone in five limbs (20%), and combined tibial and femoral osteotomies in 10 limbs (40%). Sixteen limbs (64%) had genu varum, and 9 limbs (36%) had genu valgum deformities. The average follow-up period was 28 months (range: 24–36), during which no patients were lost. The osteotomy achieved clinical union in 3.7 ± 0.9 months (median 4, range 2–6).

**Table 1:** Patients’ Data

		Count	%
Gender	female	10	50%
	male	10	50%
Side	left	10	40%
	right	15	60%
Limb alignment	varus	16	64%
	valgus	9	36%

The preoperative LD-SRS score was 3.2 ± 0.4 (median 3.2, range 2.4–3.9), which improved significantly postoperatively to 4.3 ± 0.7 (median 4.5, range 2.5–5) (*p* < 0.001) (Table 2).

**Table 2:** Results (normal range: mTFA 5º-9º, mMPTA 85º-90º, mLDFA 85º-90º, MAD 0±3 mm)

	Pre-op	2-year Post-Operative	*P*-Value
LD-SRS Score	Mean 3.2 ± 0.4 Median 3.20 (2.35–3.85)	Mean 4.3 ± 0.7 Median 4.46 (2.5 − 5)	< 0.001*
mTFA (degrees) in varus group	Mean 27.8 ± 12.2 Median 28 (11–46)	Mean 3.4 ± 6.4 Median 3.5 (9 − 14)	< 0.001*
mMPTA (degrees) in varus group	Mean 78.7 ± 8 Median 79.5 (65–95)	Mean 88.6 ± 3.4 Median 88 (80–95)	< 0.001*
mLDFA (degrees) in varus group	Mean 102.4 ± 9.7 Median 104.5 (85–122)	Mean 89.9 ± 3.1 Median 90 (82–95)	< 0.001*
MAD (mm) in varus group	Mean 82.2 ± 35.6 medial Median 86.5 (23–137)	Mean 9.2 ± 20.1 medial Median 6 (33 valgus–39 varus)	< 0.001*
mTFA (degrees) in valgus group	Mean17.3 ± 7.9 Median 19 (5–29)	Mean 2 ± 5.2 valgus Median 0 (15 valgus − 2 varus)	0.008*
mMPTA (degrees) in valgus group	Mean 89.7 ± 3.9 Median 88 (85–96)	Mean 88.2 ± 2.9 Median 88 (85–93)	0.385
mLDFA (degrees) in valgus group	Mean 77.9 ± 9.8 Median 79 (61–89)	Mean 88.1 ± 3.1 Median 87 (84–94)	0.027*
MAD (mm) in valgus group	Mean 41.8 ± 22.2 lateral Median 37 (21–82)	Mean 4.6 ± 12.6 lateral Median 0 (37 valgus − 4 varus)	0.008*

**Fig. 2:**
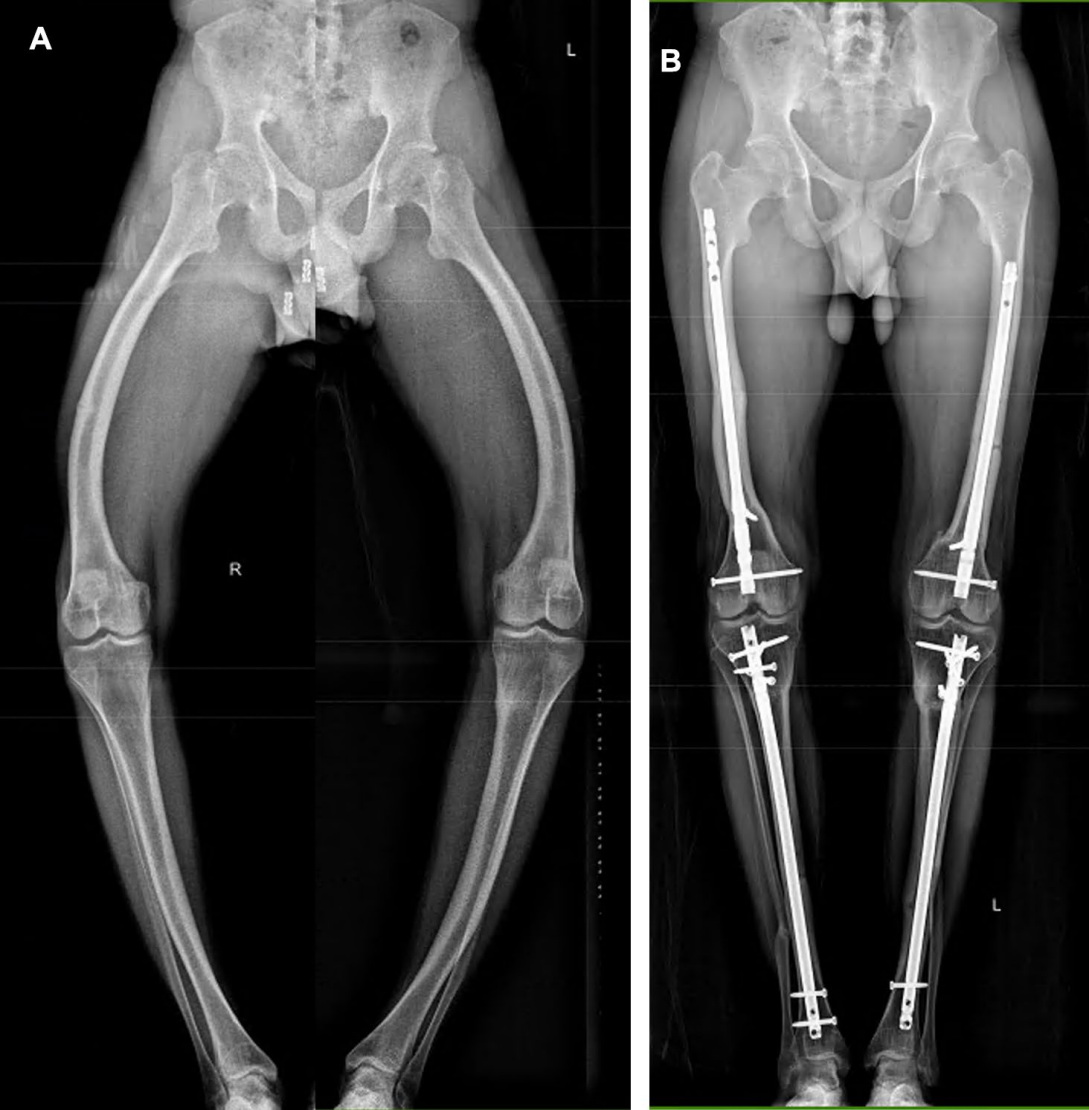
a case of varus alignment with greater femoral varus; A: pre-op standing full-length radiographs, B: full-lengthen radiographs at final fallow-up

**Fig. 3:**
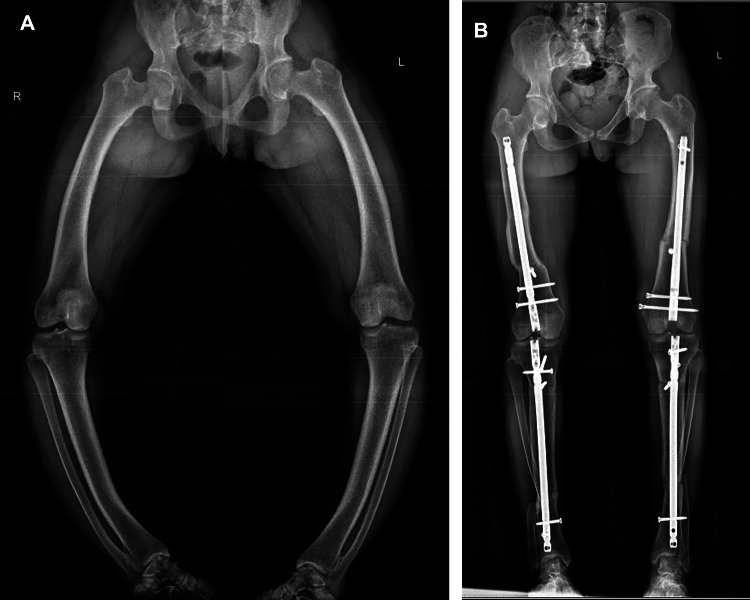
case of varus alignment where femur and tibia equally contribute to the deformity; A: pre-op standing full-length radiographs, B: full-lengthen standing radiographs at final fallow-up

In patients with genu varum deformity (16 limbs) (Figs. 2 and 3), the average preoperative mTFA was 27.8° ± 12.2 ° varus alignment (median 28°, range 11–46°), which improved to 3.4° ± 6.4° (median 3.5°, range 9–14°) at final follow-up (*p* < 0.001). The average preoperative mLDFA was 102.4° ± 9.7° (median 104.5°, range 85–122°), which improved to 89.9° ± 3.1° (median 90°, range 82–95°) at final follow-up (*p* < 0.001). The average preoperative mMPTA was 78.7° ± 8° (median 79.5°, range 65–95°), which improved to 88.6° ± 3.4° (median 88°, range 80–95°) at final follow-up (*p* < 0.001). The average preoperative MAD was 82.2 ± 35.6 mm medial (median 86.5 mm, range 23–137 mm), which improved to 9.2 ± 20.1 mm medial (median 6 mm, range 33 valgus –39 varus mm) at final follow-up (*p* < 0.001). At the final follow-up, limb alignment was Zone 1 in eight limbs, Zone 2 in eight limbs, and no limbs were in Zone 3.

**Fig. 4:**
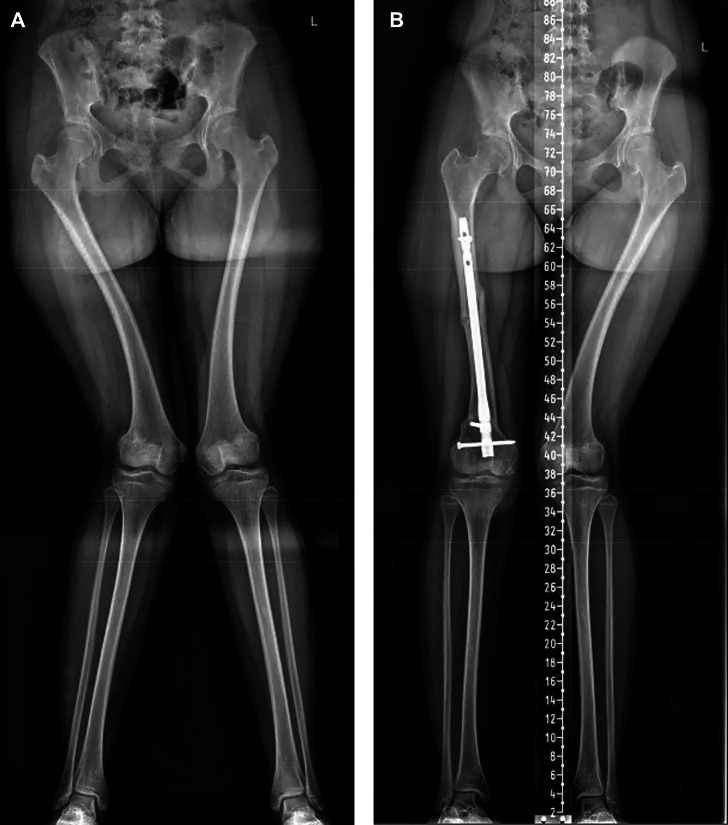
a case of valgus alignment that is femoral in origin; A: pre-op standing full-length radiographs, B: full-lengthen standing radiographs at final fallow-up

**Fig. 5:**
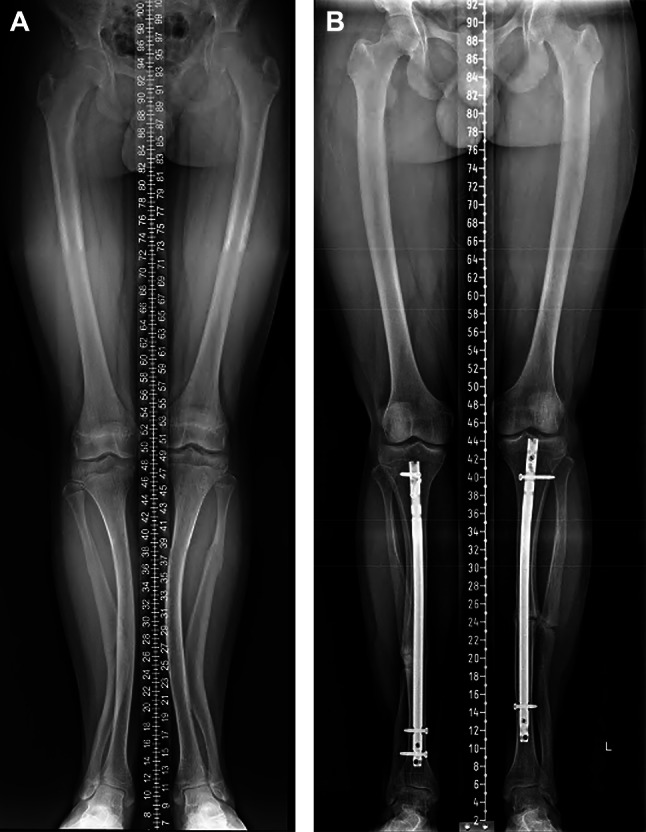
a case of valgus alignment that is tibial in origin; A: pre-op standing full-length radiographs, B: full-lengthen standing radiographs at final fallow-up

In the genu valgum group (nine limbs) (Figs. 4 and 5), the average preoperative mTFA was 17.3° ± 7.9° valgus alignment (median 19°, range 5–29°), which improved to 2° ± 5.2° valgus alignment (median 0°, range 15° valgus to 2° varus) at final follow-up (*p* = 0.008). The average preoperative mLDFA was 77.9° ± 9.8° (median 79°, range 61–89°), which improved to 88.1° ± 3.1° (median 87°, range 84–94°) at final follow-up (*p* = 0.027). The average preoperative mMPTA was 89.7° ± 3.9° (median 88°, range 85–96°), which improved to 88.2° ± 2.9° (median 88°, range 85–93°) at final follow-up (*p* = 0.385, non-significant). The average preoperative MAD was 41.8 ± 22.2 mm lateral (median 37 mm, range 21–82 mm), which improved to 4.6 ± 12.6 mm lateral (median 0 mm, range 37° valgus alignment to 4° varus alignment) at final follow-up (*p* = 0.008). At the postoperative follow-up, limb alignment was Zone 1 for eight limbs, Zone 3 for one limb, and no limbs were in Zone 2.

It is worth noting that the final alignment of some patients was Zone 2 (eight patients) or Zone 3 (one patient), which was not the intended correction. This was attributed to their soft bone, which led to some loss of correction during follow-up; however, all patients had a Zone 3 preoperative limb alignment, and they all had a significant percentage of their deformity corrected.

Average time till patients were comfortable to bear weight was 7 (1–14) days, all patients had full knee range of motion able to extend fully and flex to 130 degrees pre-operatively; post-operatively all patients were able to extend their knees fully and their average knee flexion range of motion was 120 (100–130) degrees.

### Complications

The Clavien–Dindo–Sink classification [22] of orthopedic surgical complications was used to evaluate complications. Only two limbs developed knee stiffness (Type III complication), which improved after manipulation under anesthesia. No infection, vascular or neurological complications, or nonunion occurred.

## Discussion

Lower limb deformities lead to early osteoarthritis of the weight-bearing joints as well as psychological and social disabilities. The aim of deformity correction is to restore joint orientation angles within the normal anatomical range to prevent the development of early osteoarthritis [23].

Deformities due to metabolic bone diseases usually occur in multiple limb segments, with a tendency for recurrence if the disease is not metabolically controlled [24].

Stevens et al. [24, 25] reported good outcomes in correcting deformities in patients with X-LHPR using guided growth in a group of patients younger than 10 years. A similar study by Horn et al. [4] concluded that guided growth could be effective in patients with more than 3 years of growth remaining. For those older patients, deformity correction by osteotomy would be a better plan; however, in metabolic bone diseases, osteotomies tend to heal over a prolonged period, with an increased risk of nonunion and recurrence. Choi et al. [26] recommended performing osteotomies on patients with X-LHPR only if their blood phosphorus level was > 2.5 mg/dL to avoid delayed union. In our study, these recommendations were adhered to, and no cases of delayed union or nonunion were encountered.

Both external and internal fixation can be used to correct such complex deformities. External fixation is less invasive, adjustable, versatile, and more accurate, but it is cumbersome for the patient. Its complications include pin tract infection and joint stiffness [27]. Although internal fixation is more comfortable for the patient, it is technically demanding and non-adjustable after surgery; therefore, there is a risk of less accurate correction [29].

In 1994, Stanitski [27] first reported the use of the Ilizarov technique for treating deformities specifically associated with X-LHPR. Treatment involved 18 limb segments in eight patients. The total time in the external fixator averaged 12 weeks, and the only reported complications were pin tract infections and mild translational deformities in two patients. Emphasis was placed on the slow correction of deformity, with incremental opening of osteotomies at a rate no faster than 0.5 mm per day; follow-up was only 1 year.

Kanel and Price [28] reported on a series of nine children with X-LHPR; most underwent low-energy, acute, corrective osteotomies and fixation, and all were treated with a unilateral external fixator. The only notable complication was the development of compartment syndrome in one patient, necessitating a fasciotomy. In our study, prophylactic subcutaneous leg fasciotomy with acute tibial correction was routinely performed.

In metabolic conditions with soft bone, fixation with IMNs stabilizes the full length of the bone, which can prevent the recurrence of deformities that may occur with metabolic decompensation in the postoperative period [29, 30]. The emergence of the FAN technique combines the advantages of both methods—namely, the accuracy of deformity correction using an intraoperative fixator, followed by intramedullary nailing to maintain the achieved alignment. This method provides stability, patient convenience, and prevents recurrence of the deformity.

In a study conducted by Eralp et al. [31], they compared FAN with circular external fixator treatment for bone deformities. No significant differences were found in the accuracy of deformity correction. The authors concluded that FAN was more acceptable to patients and that the retained intramedullary nail may protect against recurrent deformities. Moreover, in the circular external fixator group, six patients (66%) had pin tract infections.

Another technique is fixator-assisted plating, described by Rozbruch et al. [32]. Although locking plates provide greater mechanical rigidity at the osteotomy site, common disadvantages include risk of implant failure, and potential bone loss under the plate. In addition, their use is challenging when multiple osteotomies are needed because extensive soft tissue stripping is required, and the entire length of the bone is usually not spanned by the plate. A common problem with fixator-assisted plating is painful hardware, which often necessitates implant removal, as reported by Rozbruch et al. [32]. The use of an IMN avoids this problem, and the retained IMN protects against deformity recurrence.

In metaphyseal osteotomies, which are typically necessary because most cases have a primary deformity apex in the metaphyseal region, careful preoperative planning and precise use of blocking screws ensure accurate correction.

This study has limitations, including being conducted in a single center, with all surgeries performed by one surgeon, and the lack of a comparative group. Additionally, a longer follow-up period is recommended to assess the rate of deformity recurrence following this technique and to determine whether implants should be removed.

In conclusion, correcting severe lower limb frontal plane deformities in adolescents with hypophosphatemic rickets using IMNs yields good 2-year postoperative clinical and radiographic outcomes.

## Supplementary Information

Below is the link to the electronic supplementary material.

Supplementary Material 1

Supplementary Material 1

## Data Availability

No datasets were generated or analysed during the current study.
