# Supplementary figures and images for: Clinical and radiological outcomes of using locked intramedullary nails in the treatment of severe frontal plane lower limb deformity in adolescents with hypophosphatemic rickets (mid-term results)
Source: Arch Orthop Trauma Surg. 2026 May 4;146(1):174. doi: 10.1007/s00402-026-06313-4 (PMC13139209; doi:10.1007/s00402-026-06313-4)

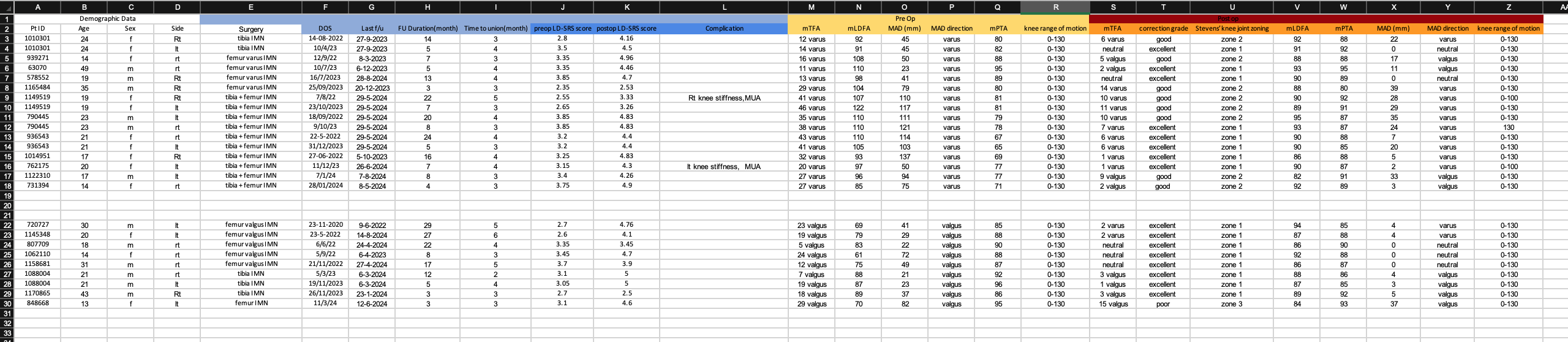

Supplement: Supplementary file 2 — Supplementary Material 1 [file 402_2026_6313_MOESM2_ESM.png]
